# Identifying *Group-Specific* Sequences for Microbial Communities Using Long *k*-mer Sequence Signatures

**DOI:** 10.3389/fmicb.2018.00872

**Published:** 2018-05-03

**Authors:** Ying Wang, Lei Fu, Jie Ren, Zhaoxia Yu, Ting Chen, Fengzhu Sun

**Affiliations:** ^1^Department of Automation, Xiamen University, Xiamen, China; ^2^Molecular and Computational Biology Program, University of Southern California, Los Angeles, CA, United States; ^3^Department of Statistics, University of California, Irvine, Irvine, CA, United States; ^4^Bioinformatics Division, Tsinghua National Laboratory of Information Science and Technology, Tsinghua University, Beijing, China; ^5^Department of Computer Science and Technology, Tsinghua University, Beijing, China; ^6^Center for Computational Systems Biology, Fudan University, Shanghai, China

**Keywords:** long *k*-mer, classification, *group-specific* sequence, metagenomics, microbial community, disease prediction

## Abstract

Comparing metagenomic samples is crucial for understanding microbial communities. For different groups of microbial communities, such as human gut metagenomic samples from patients with a certain disease and healthy controls, identifying *group-specific* sequences offers essential information for potential biomarker discovery. A sequence that is present, or rich, in one group, but absent, or scarce, in another group is considered “*group-specific*” in our study. Our main purpose is to discover *group-specific* sequence regions between control and case groups as disease-associated markers. We developed a long *k*-mer (*k* ≥ 30 bps)-based computational pipeline to detect *group-specific* sequences at strain resolution free from reference sequences, sequence alignments, and metagenome-wide *de novo* assembly. We called our method MetaGO: *Group-specific* oligonucleotide analysis for metagenomic samples. An open-source pipeline on *Apache Spark* was developed with parallel computing. We applied MetaGO to one simulated and three real metagenomic datasets to evaluate the discriminative capability of identified *group-specific* markers. In the simulated dataset, 99.11% of *group-specific* logical *40*-mers covered 98.89% *disease-specific* regions from the disease-associated strain. In addition, 97.90% of *group-specific* numerical *40*-mers covered 99.61 and 96.39% of differentially abundant genome and regions between two groups, respectively. For a large-scale metagenomic liver cirrhosis (LC)-associated dataset, we identified 37,647 *group-specific 40-*mer features. Any one of the features can predict disease status of the training samples with the average of sensitivity and specificity higher than 0.8. The random forests classification using the top 10 *group-specific* features yielded a higher AUC (from ∼0.8 to ∼0.9) than that of previous studies. All *group-specific 40-*mers were present in LC patients, but not healthy controls. All the assembled 11 *LC-specific* sequences can be mapped to two strains of *Veillonella parvula*: UTDB1-3 and DSM2008. The experiments on the other two real datasets related to Inflammatory Bowel Disease and Type 2 Diabetes in Women consistently demonstrated that MetaGO achieved better prediction accuracy with fewer features compared to previous studies. The experiments showed that MetaGO is a powerful tool for identifying *group-specific k*-mers, which would be clinically applicable for disease prediction. MetaGO is available at https://github.com/VVsmileyx/MetaGO.

## Introduction

High-throughput sequencing technologies have ushered in new views of ubiquity and diversity of microbial communities (Yatsunenko et al., 2012). Metagenomic sequencing data permit comprehensive profiling of microbial communities at single-nucleotide resolution. The ability to compare two groups of metagenomic samples is crucial for understanding microbial communities and their effects on hosts. Typically, for two groups of individuals, patients with a certain disease and healthy individuals, *group-specific* markers offer significant support in understanding and predicting disease. Here, “*group-specific* markers” can be genes, species, or sequences present, or rich, in one group, but absent, or scarce, in another group. “*Group-specific*” focuses on the highest discriminative power, rather than the statistically significant difference (White et al., 2009; Segata et al., 2011), to classify, or predict, case and control groups. Accordingly, prediction performance evaluates the discriminative capability of identified *group-specific* features.

Some studies characterized microbiomes by aligning reads to reference genomes or 16S rRNA marker genes (Costello et al., 2009; Quast et al., 2012; Lozupone et al., 2013; Jiang, 2015). It was realized that the alignment-based methods were limited by incomplete or inaccurate reference sequences (Kunin et al., 2008). For example, only about 31.0–48.8% of the shotgun reads from human gut could be aligned to 194 public human gut bacterial genomes, and 7.6–21.2% to the bacterial genomes deposited in GenBank (Qin et al., 2010). Recently, more studies adopted reference-free strategies to analyze the compositional differences of metagenomes between control and case groups at the microbial gene, gene set, or species levels. Generally, contigs were produced through the metagenome-wide *de novo* assembly, and a gene catalog was established through open-reading frame (ORF) prediction. The above processing was first applied to human microbiome of inflammatory bowel disease (IBD) (Qin et al., 2010). Follow-up investigations were conducted based on the constructed gene sets: approximately 60,000 associated gene markers were identified to predict Type 2 Diabetes (T2D), and the concept of a metagenomic linkage group was proposed, which is a group of genes that co-exist among samples and has a consistent abundance level and taxonomic assignments (Qin et al., 2012). The metagenomic gene clusters based on high abundance correlations were further applied to predict T2D in European women using gut metagenomic samples (Karlsson et al., 2013). The gene clusters containing a large number of genes (such as >700) assist *de novo* genome assembly to discover microbial species associated with liver cirrhosis (LC) (Qin et al., 2014) and IBD (Nielsen et al., 2014). Pasolli et al. (2016, 2017) conducted prediction tasks on 2424 metagenomic samples from eight large-scale projects using species-level relative abundances and the presence of strain-specific markers as features. Wen et al. (2017) compared the predicting performances of three types of biomarkers: sequenced reference genomes, genes and gene clusters, for ankylosing spondylitis based on gut metagenomic samples. They found that gene markers performed better than reference genome markers and clustered gene markers, and the clustered gene markers might be limited by the unknown taxonomic organisms in clusters. Almost all the above studies followed the analysis pipeline of *de novo* contig assembly, gene prediction, and gene clustering. Previous studies concluded that metagenome-assembly performs well for microbial communities that have high coverage of phylogenetically distinct, and low taxonomic diversity (Papudeshi et al., 2017), but the presence of closely related strains in one community would substantially have negative effect on the assembly performance (Sangwan et al., 2016; Sczyrba et al., 2017). Moreover, high co-abundance among species would result in multiple species in one cluster (Nielsen et al., 2014). Therefore, components with closely related genome sequences or abundance would diminish the performance of assembly and clustering in microbial community studies.

Besides genes or species, assembled contigs have also been used as features to predict disease. Several contig binning tools, such as CONCOCT (Alneberg et al., 2014), MaxBin2.0 (Wu et al., 2016), COCACOLA (Lu et al., 2017), and MetaGen (Xing et al., 2017), were developed for binning contigs assuming that contigs with similar coverage/relative abundances over different samples come from the same genomes. In particular, although the main purpose of MetaGen (Xing et al., 2017) is to identify microbial species in the community through binning, the study not only designed comprehensive experiments to analyze the effect of sequencing depth, sample size, number of species and sequence similarity, but also used the relative abundance of each bin to predict IBD/T2D/obesity disease on metagenomic datasets to evaluate the binned microbial composition. Similarly, Ren et al. (2017) developed a novel pipeline to predict the disease status of LC using the abundance of viral contig bins. Both studies made novel attempts to identify markers through assembling *de novo* reads into contigs and then binning contigs, which achieved excellent predicting results. The basic idea is to discover species markers that are differentially abundant between case and control groups. However, current assembly tools are hard to handle large-scale datasets: reads assembly involves the construction of *De Bruijn* graph, error correction, and path resolution; contig binning requires mapping reads to the assembled contigs; both would require extremely large memory and are very time-consuming. Also, if the main purpose is to discover *group-specific* markers, it is not necessary to assemble contigs for the genomes that are not associated with the disease.

The *k*-mer frequencies (i.e., the number of occurrences of *k*-mers within the whole sequencing data) are another representative alignment-free feature to characterize a microbial community. The frequency distributions of *2–10*-mers were used to compare metagenomic and meta-transcriptomic communities (Jiang et al., 2012; Wang et al., 2014; Liao et al., 2016) or to improve contig binning within a community (Wang et al., 2017). Also, Cui and Zhang (2013) classified clinical metagenomic samples using the frequencies of *2–10*-mers.

However, *2–10*-mers are too short to capture specific details inside the microbial community, such as sequences present, or rich, in one group, but absent, or scarce in another group. Intuitively, longer *k*-mers contain richer biological information in the nucleotide sequences. The long *k*-mers had been mainly utilized as seed index in sequence assembly and alignment (Li et al., 2010; Grabherr et al., 2011). Recently, long *k*-mers (≥20 bp) began to be utilized to more applications: our previous study explored the effect of *k*-mer length on an unsupervised comparison between metagenomic samples and verified the promising performance of long *k*-mers to depict the specific characteristics of microbial communities (Wang et al., 2015). Han et al. (2017) detected differentially abundant *21*-mers in metagenomic samples from T2D and healthy individuals, assembled the reads containing those *21*-mers into contigs, and then predicted genes based on the contigs. Finally, they used the gene abundances to predict T2D status. Our study differs from Han et al. (2017) in the sense that we do not predict genes based on the contigs assembled from reads containing statistically differentially abundant *k*-mers. Instead, we identified *group-specific k*-mers using discriminative power to separate two groups and predicted disease status with *k*-mers as features. Moreover, *group-specific k*-mers were assembled to contigs directly. Rahman et al. (unpublished) found significant differentially abundant *31*-mers between two groups of 1000 genomes data and discovered SNPs between different populations, which is highly different from the objectives of this study. The frequency vector of long *k*-mers (∼30 bp) was also applied to calculate the dissimilarity between metagenomic samples using 16 standard ecological distances (Benoit et al., 2016). The long *k*-mers began to present attractive potentials to characterize high-throughput sequencing data.

Since sufficiently long *k*-mers are usually specific to a genome (Fofanov et al., 2004), therefore, we proposed a computational framework to identify *group-specific* sequences between two groups of metagenomic samples with long (≥30 bp) *k-*mers in this study. We call our method MetaGO: *Group-specific* oligonucleotide analysis for metagenomic samples. The main purpose of MetaGO is to discover *group-specific* sequence regions between control and case groups as disease-associated markers. Instead of using statistically significant difference as index, we considered the discriminant power to separate two groups of single *k*-mer. A *k*-mer is considered *group-specific* if (1) the average of sensitivity and specificity (ASS) is higher than a preset threshold when using the presence/absence of the *k*-mer on the sequencing data to predict disease status, or (2) the *k*-mer’s frequencies are significantly different between two groups of samples (Wilcoxon rank-sum test, *p*-value ≤ 0.01) and the ASS is higher than a preset threshold using logistic regression. The *group-specific k*-mers are identified based on the training set. In our study, *k*-mer length is set between 30 and 40 given the tradeoff among sensitivity, specificity, and computational cost. To reduce the computational burden from long *k*-mers, we developed an open-source, parallel-computing pipeline on *Apache Spark*. Once the *group-specific k-*mers are identified, we assembled them into *group-specific* sequences. The assembly on the markedly reduced number of long *k*-mers will be more computationally efficient and accurate.

MetaGO was tested on one simulated and three real metagenomic datasets. In the simulated dataset, for the two strains sharing 87% common sequences where one is disease specific and the other one is present in both groups, we identified *group-specific* logical *40*-mers that covered 98.89% (recall) of the *disease-specific* sequence regions from the disease-associated strain with 98.91% precision. In addition, 98.83% of the *group-specific* numerical *40*-mers covered 99.01 and 97.30% of the differential-abundant genome and regions, respectively. For the metagenomic LC-associated dataset (Qin et al., 2014), it is composed of human fecal samples from 98 LC patients and 83 healthy controls, as well as an additional independent dataset containing 25 patients and 31 controls. The *k*-mer length was set as 40 because of the large sample size (number of samples). In our experiment, two-thirds of the 98 patients and 83 control samples were randomly selected as the training set, leaving one-third as the validation set and the extra 25 patients and 31 controls as the independent testing set. In total, 37,647 *group-specific 40*-mers were identified on the training set, and 35,652 and 12,944 of the *group-specific 40*-mers yielded ASS ≥ 0.8 on the validation and testing sets, respectively. The *single-logical-feature* predictor with the highest ASS score 0.87 on the training set predicted the disease status in the validation and testing sets with ASS score as 0.88 and 0.83, respectively. Using the top 10 *group-specific 40*-mers, the random forests classifier achieved the area under the receiver operating characteristic (AUC) as 0.963, 0.969, and 0.942 on training, validation, and testing sets, respectively. It is interesting to note that all 37,647 *40-*mers were present in LC patients but absent from healthy controls. The *LC-specific 40*-mers were assembled into 11 sequences with a length between 210 and 350 bp, and they demonstrated the distinguishing coverages between two groups. All the identified *LC-specific* sequences could be matched to two strains of *Veillonella parvula*, UTDB1-3 and DSM2008 with 97–100% identity. And 83.2 and 79.6% of the 37,647 *group-specific 40*-mers could be matched to strain UTDB1-3 and DSM2008, respectively.

We also identified *group-specific k*-mers based on two more metagenomic disease-associated datasets: IBD associated (Qin et al., 2010) and WT2D (T2D in women) associated (Karlsson et al., 2013). Based on the identified *group-specific k*-mers, our pipeline achieved substantially better prediction performance using relatively fewer features compared with previous studies having identical or relaxed experimental settings. All experiments demonstrated long *k*-mers to be more efficient in capturing the specific information of sequencing data and discriminating gut microbiome communities between control and case groups. It should be noted that *group-specific* sequences are identified free from reference sequences, metagenome-wide assembly, and sequence alignments. MetaGO greatly facilitates the identification of clinically meaningful biomarkers.

## Materials and Methods

### Description of Terms

*A group-specific feature* is a *k*-mer present, or rich, in the metagenomic sequencing data of one group, but absent, or sparse, in the sequencing data of another group. A *k-*mer is a word composed of *k* oligonucleotides, and the total number of all possible *k*-mers is 4^k^.

We defined *k*-mer features in the following two ways:

*Numerical features* are the normalized frequencies of *k*-mers. The numerical feature of a *k*-mer *i* in sample *j* is denoted as *f_i_*(*j*) and is defined in Equation (1), where f_i_°(j) is the number of occurrences of *k*-mer *i* in sample *j*, and *n* is the total number of *k*-mers, that is 4*^k^*. So the normalization is the number of occurrences of the *k*-mer over the total number of occurrences for all *k*-mers in one sample. Each *k*-mer has the same length *k*, so length is not considered during the normalization.

(1) $$f_{i}(j)=\frac{f_{i}^{{}^{\circ}}(j)}{\sum_{i=1}^{n}f_{i}^{{}^{\circ}}(j)},i=1,2,...,n.$$

*Logical features* are the logicalization of numerical features. They use 1 and 0 to represent *k*-mers as present or absent in one sample, as shown in Equation (2),

(2) $$f_{i}^{\left(l\right)}(j)=\left\{ \begin{array}{l} 1\;\text{if}\;f_{i}(j)>0\\ 0\;\text{if}\;f_{i}(j)=0 \end{array}\right.,$$

where f_i_^(l)^(j) is the logical value of *k*-mer *i* in sample *j*, and the superscript “*l*” indicates logical feature.

*A single-logical-feature predictor*, as represented in Equations (3) and (4), is used to predict disease status based on whether a *k*-mer *i* is present in the sequencing data of sample *j* or not.

(3) $$f_{i}^{\left(l\right)}(j)=\left\{ \begin{array}{l} 1\;\text{then sample }j\in \text{Group}+\\ 0\;\text{then sample }j\in \text{Group}- \end{array}\right.$$

or

(4) $$f_{i}^{\left(l\right)}(j)=\left\{ \begin{array}{l} 1\;\text{then sample }j\in \text{Group}-\\ 0\;\text{then sample }j\in \text{Group}+ \end{array}\right..$$

*A single-numerical-feature logistic regression* predicts the case and control status based on one single numerical feature, and it is used as the independent variable in a logistic regression. An example of each term above is given in **Supplementary File S1**.

### The Computational Framework to Identify *Group-Specific* Sequences

**FIGURE 1:**
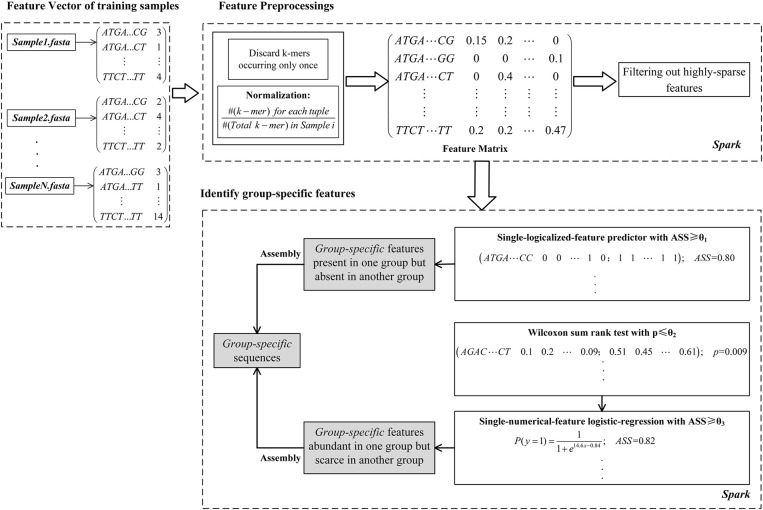
The MetaGO framework to identify *group-specific* sequences with long *k*-mer features. The framework is composed of three modules. (1) The feature vector of each metagenomic sample is composed of the frequencies of all *k*-mers. (2) The *k*-mers are preprocessed by discarding features occurring only once, normalization, integrating the matrix and removing the *k*-mers absent from most training samples. (3) The features are represented as logical and numerical forms, and the features with high discriminant power are identified to be *group-specific*.

As shown in **Figure 1**, the computational framework of MetaGO consists of three modules. (1) *Creating a feature vector for each sample*. The feature vector is composed of the number of occurrences for each *k*-mer through all reads in one sample. (2) *Feature preprocessing*. After removing *k*-mers occurring only once and normalizing *k-*mer frequencies, the feature matrix is integrated on the feature vectors across all training samples. The *k*-mers that are absent in most training samples are filtered out. (3) *Identifying group-specific features*. The logical and numerical features with high discriminant power are selected.

MetaGO was developed on *Apache Spark* to reduce computational costs through parallel running on HDFS of Hadoop or a stand-alone multi-core server. The open-source pipeline is available at https://github.com/VVsmileyx/MetaGO.

### Module 1: Creating Feature Vectors

A feature vector consists of elements that account for the number of occurrences (i.e., frequency) for each *k*-mer through all the reads in one metagenomic sample. Existing tools, such as DSK (Rizk et al., 2013) or JELLYFISH (Marçais and Kingsford, 2011), are available for counting *k*-mer frequency. In our study, we used DSK to count *k*-mers. The reverse complements of reads were taken into consideration. A *k*-mer and its reverse complement were considered as the same object, so the theoretical dimension of a feature vector for one sample is shrunk to $\frac{4^{k}+2^{k}}{2}$ for even *k* and $\frac{4^{k}}{2}$ for odd *k*. Furthermore, only the *k*-mers that occur in a sample are stored in the feature vector to reduce storage space.

### Module 2: Feature Preprocessing

#### Discard *k-*mer Features Occurring Only Once

With the increase of *k*-mer length, *k*-mer frequency decreases exponentially, and the *k*-mer vector is highly sparse. A *k*-mer occurring only once might be caused by low abundance or sequencing errors. To achieve reproducible and stable prediction models, *k*-mers occurring once were removed from the frequency vector, and this step was implemented by DSK during *k-*mer counting in our study.

#### Normalize *k*-mer Frequencies

Owing to different sequencing depths in samples, the frequency of a *k*-mer is normalized using Equation (1) by the total number of occurrences of all *k*-mers.

#### Build Feature Matrix Across Training Samples

Feature vectors across all training samples are integrated as a matrix. This step is extremely time- and memory-consuming as a result of the large sample size and the long *k*-mer length. Just storing non-zero *k*-mers in each feature vector, the integration process requires huge amounts of sorting and matching of *k*-mers. When *k* = 40, approximately 10^9^ *40*-mer features occur more than once. The feature matrix *F* is denoted as Equation (5), where *k*-mer_1_, *k*-mer_2_, …, *k*-mer*_m_* are the *m k*-mer features, and *S*_1_, *S*_2_, …, *S_N_* are the *N* training samples from case and control groups.

(5) $$\begin{array}{cc} & \begin{array}{cccc} \;\;S_{1}\;\; & \;\;S_{2}\; & \cdots \;\; & \;S_{N}\; \end{array}\\ \begin{array}{r} \begin{array}{c} \begin{array}{l} \begin{array}{r} k-{\text{mer}}_{1}\\ F=k-{\text{mer}}_{2}\\ \vdots \\ k-{\text{mer}}_{m} \end{array} \end{array} \end{array} \end{array} & \left(\begin{array}{cccc} f_{1}(1) & f_{1}(2) & \cdots & f_{1}(N)\\ f_{2}(1) & f_{2}(2) & \cdots & f_{2}(N)\\ \vdots & \vdots & \vdots & \vdots \\ f_{m}(1) & fm(2) & \cdots & f_{m}(N) \end{array}\right) \end{array}\;\;\;\;\;\left(5\right)$$

#### Remove Highly-Sparse Features

The “highly-sparse” feature means that a *k*-mer is absent in most training samples, i.e., the frequencies of *k*-mers are 0 in most training cases and controls. Such features have limited contributions to classification. In our study, if a *k-*mer is absent in more than 80% of control samples and 80% of case samples, the feature is removed. The stringent threshold of 80% offers high confidence in filtering out less useful features.

### Module 3: Identifying *Group-Specific* Features

After preprocessing, about 10^6^ features still remain for *40*-mers. Simple feature-ranking filtering is more suitable than Wrapper feature selection. Wrapper methods consider the selection of a set of features as a search problem in which different combinations are prepared, evaluated, and compared to other combinations. The dimension of combination space is extremely high for a large number of features in our study. The filtering of *k*-mers is only based on the training data without touching the validation and testing data.

#### Identify *Group-Specific* Logical Features Based on a *Single-Logical-Feature* Predictor

**FIGURE 2:**
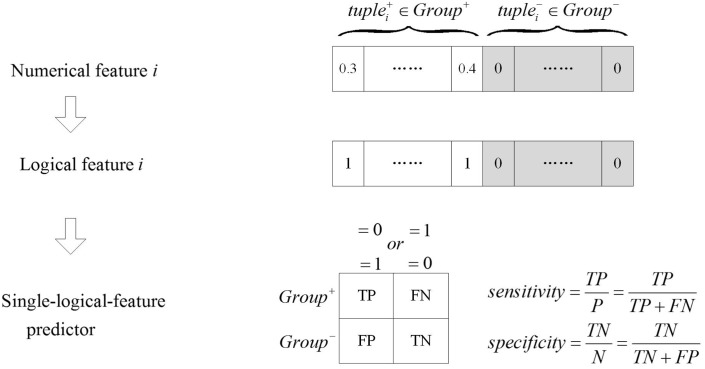
The *single-logical-feature* predictor. The numerical feature is transformed into the logical feature. Based on the logical value of the feature, the *single-logical-feature* predictor is designed, and the corresponding ASS is calculated.

As shown in **Figure 2**, numerical features were transformed to logical features using Equation (2), and the *single-logical-feature* predictors were created according to Equations (3) or (4). The performance of a predictor was evaluated by ASS, an average of sensitivity and specificity. If a *single-logical-feature* predictor achieves ASS ≥ 𝜃_1_, the corresponding *k*-mer is identified to be *group specific*. The *group-specific* logical features are present in one group but absent in another group.

In our study, *𝜃*_1_ was set as 0.80, which means that each *group-specific k*-mer alone can separate two groups of training samples with ASS ≥ 0.8 solely. Some researchers would prefer a statistical test, such as Chi-squared test, to rank the features. To accommodate this preference, we calculated *p*-values of Chi-squared test for the same feature set. Among the two feature lists with the 400 largest ASS values and the 400 smallest *p*-values, 392 features were present in both lists in the same order. Therefore, both ASS and Chi-squared test provide consistent ranks of the features. In our pipeline, users have the option to choose either ASS or Chi-squared test as evaluation metrics.

#### Identify *Group-Specific* Numerical Features Based on a *Single-Numerical-Feature* Logistic-Regression Predictor

First, Wilcoxon rank-sum test is applied to the numerical features to select *k*-mers with differential abundance (*p*-value ≤ 𝜃_2_) between two groups. However, our main goal is to identify features with the most discriminant power. Therefore, we fit logistic regression for each numerical *k*-mer feature that passed the Wilcoxon rank-sum test over all the training samples, and we term this as *single-numerical-feature* logistic-regression predictor. We used ASS ≥ 𝜃_3_ as a metric to identify *group-specific* numerical *k*-mers. In our study, we used 𝜃_2_ = 0.01 and 𝜃_3_ = 0.8

#### Random Forests Prediction of Disease Status With the Combination of Multiple Features

The *single-logical-feature* predictor and *single-numerical* logistic-regression predictor are the classifiers based on a single *k*-mer feature. Because of the complicated association between human microbiome and disease, classifiers using multiple features are expected to be more efficient than those with single features. Therefore, we used random forests to design a classifier with multiple *group-specific* features. To remove redundant features, we calculated the Pearson correlation coefficients (PCC) between the feature vectors of every pair of *k-*mers. If a pair of *k*-mers has a PCC value higher than a preset threshold, such as 0.75, one *k*-mer feature was randomly discarded. The remaining features were ranked according to the variable importance measures of Breiman’s random forests method (Breiman, 2001), and the top features were adopted to design a random forests classifier.

#### Assembly of *Group-Specific* Sequences

Using CAP3 (Huang and Madan, 1999), the identified *group-specific k*-mers based on logical and numerical features were, respectively, assembled to longer sequences. For quality control, the assembled sequences longer than a certain threshold (200 bp in our study) are considered as *group-specific* sequences.

### Parallel Computing Workflow on *Apache Spark*

The running time and memory required to integrate feature matrix and filter out less useful features expand dramatically with the increase of *k*-mer length and sample size. Fortunately, these processing steps are suitable for parallel computing. Therefore, we developed MetaGO workflow on *Apache Spark* (Zaharia et al., 2010) to implement parallel computing. *Spark* can run in local mode or cluster mode. Thus, MetaGO can run on a local stand-alone multi-core server or a distributed cluster on HDFS. The detailed description of the workflow is given in **Supplementary File S1**. The workflow is available on https://github.com/VVsmileyx/MetaGO.

### Experimental Design

#### The Setting of *k*-mer Length

A previous study showed that sufficiently long *k*-mers are usually specific to a genome (Fofanov et al., 2004). According to an observation based on 100 pairs of bacterial genomes, the average ratio of common *k*-mers between the genomes is <1.02% when *k* ≥ 30 (Le et al., 2015). Therefore, *k*-mers longer than 30 bp would possess sufficiently high sensitivity to capture the discriminate characteristics to separate two groups; thus, theoretically, longer *k*-mers are better suited to this task. At the same time, however, *k*-mer length is limited by four factors: sample size (the number of samples), sequencing depth, computational cost, and read length. First, the dimension of feature space grows exponentially with *k*. Owing to the curse of dimensionality, a limited number of samples would lead to a high false-positive rate. Therefore, a large sample size is required to obtain high specificity. Second, when sequencing depth is not deep enough to cover all the metagenomic regions, the frequencies of long *k*-mers would not be accurate. Third, with the increase of *k*-mer length, the huge number of *k*-mers leads to the explosion of memory and storage. Fourth, when the *k*-mer length is close to read length, the frequencies of *k*-mers are contaminated by the truncated sites under limited sequencing depth. Therefore, we set the *k*-mer length to be 30–40 as the reasonable tradeoff among sensitivity, specificity, and computational cost.

#### Simulated Metagenomic Dataset

**FIGURE 3:**
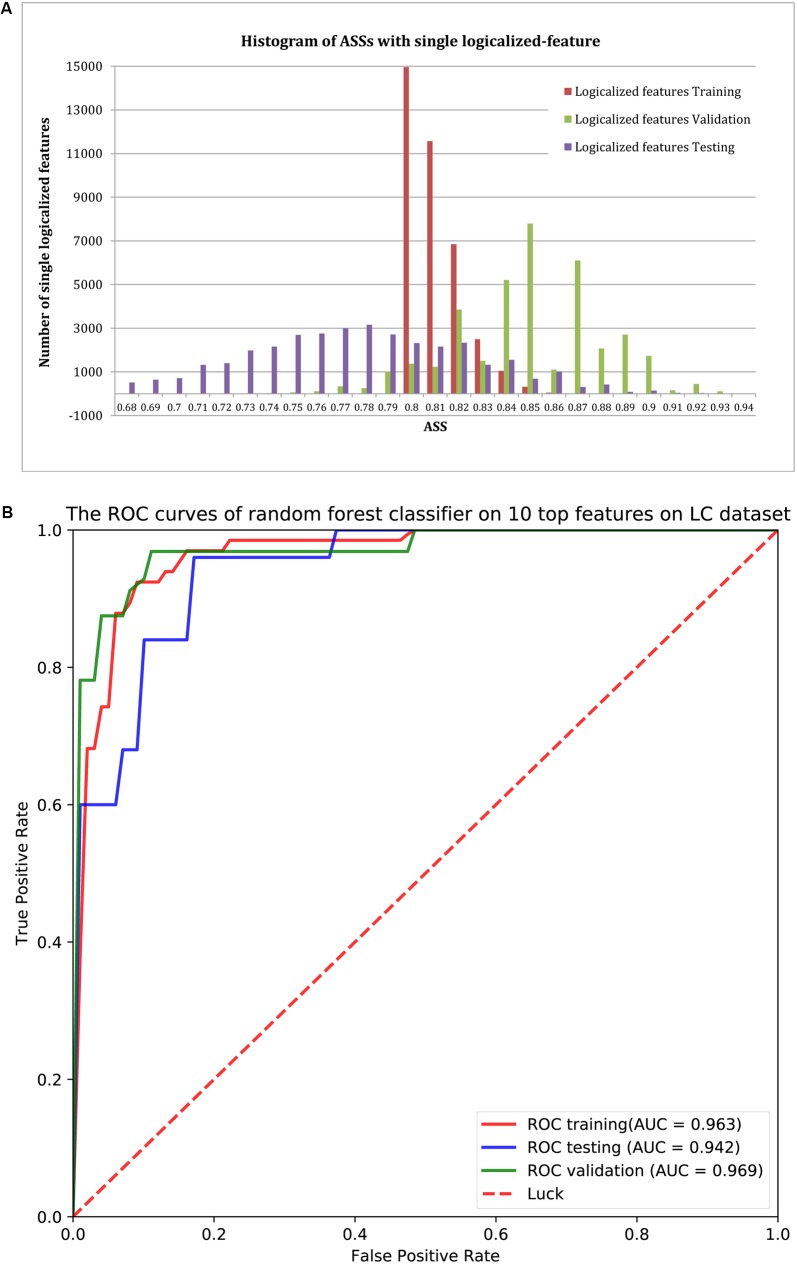
**(A)** The distribution of ASS values of the 37,302 *single-logical-feature* predictors and 345 *single-numerical* logistic-regression predictors on the identified *group-specific* features for training, validation, and testing sets. These predictors achieved better performance in the validation set compared to the training set. A total of 35,652 *group-specific* features achieved ASS ≥ 0.8 for the validation set, and 12,944 of them achieved ASS ≥ 0.8 for the testing set. **(B)** ROC curves of the random forests classifier with the top 10 features on validation and testing sets. Using the top 10 *group-specific 40*-mers, the random forests classifier achieved AUC of 0.963, 0.969, and 0.942 on training, validation, and testing sets, respectively.

Based on the relative abundances of frequent microbial genomes within human gut analyzed by Qin et al. (2010) (Figure 3 of their paper), we selected the top 10 most frequent genomes as the basis components of the simulation. The relative abundances in the control group were approximated from the medians of Figure 3 of that study (Qin et al., 2010), which were converted into the cell proportions of the 10 genomes in all the cells within the community. In addition, we added another strain *Bacteroides thetaiotaomicron* VPI-5482 to the patient group, and this strain shares about 87% common sequences with the existing *B. thetaiotaomicron* 7330. Meanwhile, we assigned Genome *Bacteroides caccae* ATCC 43185 threefold abundance in the control group than in the patient group. The remaining nine genomes have identical abundance distributions between the healthy individual and the patient groups. The detail setting is shown in **Table 1**. We used MetaSim (Richter et al., 2008) to generate 15 metagenomic samples for case and control groups, respectively. For each group, the absolute values of Gaussian noises of mean zero and standard derivation equal to each central relative abundance were added to the center relative abundance vector. Each sample contains ∼10,000,000 reads. In the evaluations, the proportion of identified *group-specific k*-mers that can be aligned to disease-specific sequence regions is called “precision,” and the proportion of disease-specific sequence regions that can be covered by *group-specific 40*-mers is called “recall.”

**Table 1:** The relative abundance profile of different genomes in control and patient groups for the simulated dataset.

Genomes	NCBI Accession ID	Relative_Abundance_H^∗^		Relative_Abundance_P^∗^
*Bacteroides thetaiotaomicron* 7330	NZ_CP012937.1		18%		
*Bacteroides thetaiotaomicron* VPI-5482	NC_004663.1	0		6%
*Bacteroides uniformis* CL03T12C37	NZ_JH724268.1		7%		
*Alistipes putredinis isolate* CAG	MNQH01000001.1		16%		
*Parabacteroides merdae* 2789STDY5834848	CZAG01000002.1		10%		
*Dorea longicatena* 2789STDY5834914	NZ_CZAY01000001.1		10%		
*Ruminococcus bromii* L2-63	FP929051.1		10%		
*Bacteroides caccae* ATCC 43185	NZ_CP022412.2	9%		3%
*Clostridium* sp. SS2/1	NZ_DS547029.1		8%		
*Eubacterium hallii isolate* EH1	NZ_LT907978.1		6%		
*Ruminococcus torques* L2-14	FP929055.1		6%		

*The relative abundances were the proportions of the number of copies of 11 genomes within the community. Bacteroides thetaiotaomicron VPI-5482 is present only in the patient group, and it is another strain of B. thetaiotaomicron. Bacteroides caccae ATCC 43185 has threefold abundance in the control group of that in the patient group. ^∗^H, healthy control; P, patient.*

#### Metagenomic Liver Cirrhosis-Associated Dataset

In recent studies, alterations in human gut microbiota have been linked to LC (Qin et al., 2014; Wiest et al., 2014). We analyzed the human fecal metagenomic samples (Qin et al., 2014) from 98 LC patients and 83 healthy controls, as well as an extra dataset composed of 25 independent patients and 31 controls. The data were sequenced with Illumina HiSeq 2000. In the experiment, two-thirds of the 98 patients and 83 control samples were randomly selected as the training set to identify *group-specific k*-mers, and the remaining one-third as the validation set. Finally, the extra 25 patients and 31 controls were applied to test the *group-specific k*-mers independently.

#### Metagenomic IBD-Associated and WT2D-Associated Datasets

The IBD dataset is composed of the human fecal metagenomic samples from 25 IBD patients and 97 controls (Qin et al., 2010). These samples were sequenced on Illumina GAIIx from the MetaHIT project (Human Microbiome Project Consortium, 2012). The WT2D dataset is composed of samples from 53 T2D patients and 43 healthy controls from European women (Karlsson et al., 2013). These samples were sequenced on Illumina HiSeq 2000. Both datasets had been predicted using various types of features (Cui and Zhang, 2013; Karlsson et al., 2013; Pasolli et al., 2016). In our study, we adopted the experimental setting of a previous study (Pasolli et al., 2016), in which 20 independent runs of 10-fold cross-validation were used to evaluate the classification.

## Results

### The Simulated Metagenomic Dataset

For logical features, there were 1,646,128 *group-specific 40*-mers using ASS ≥ 0.8 as a threshold. And 99.999% of the *40*-mers were patient specific, which means almost all the logical group-specific *40*-mers exist only in the patient group and are absent in the healthy control group. Among the logical *patient-specific 40*-mers, 99.11% of them (precision) were exactly aligned to strain *B. thetaiotaomicron* VPI-5482 (the strain present in the patient group only) and covered 98.89% (recall) of the regions that are not in the genome of the other strain *B. thetaiotaomicron* 7330. None of the *group-specific* 40-mers were aligned to *B. thetaiotaomicron* 7330, which has the same abundance on both groups. The logical *group-specific 40*-mers mainly indicate genomes present in one group but not in another group.

The remaining features were represented as numerical *40*-mers, and there were 7,891,412 *group-specific 40*-mers using *p* < 0.05 and ASS ≥ 0.8 as the thresholds. And 4,452,553 (56.42%) of them were exactly matched to *B. caccae* ATCC 43185 and covered 99.61% (recall) of the whole genome, which is differentially abundant between the healthy control and the case groups. Among the remaining *40*-mers, 3,257,251 (41.3%) of them were aligned to the common regions between *B. thetaiotaomicron* VPI-5482 and *B. thetaiotaomicron* 7330, and covered 96.39% (recall) of the common sequences. Because for the patient group, the abundance of common sequences includes VPI-5482 and *B. thetaiotaomicron* 7330, but the control group only includes *B. thetaiotaomicron* 7330, the common sequences are differentially abundant. In total, 97.72% (precision) of the identified *group-specific* numerical *40*-mers were aligned to the differentially abundant regions between the two groups.

The identified *patient-specific* and *control-specific 40*-mers from logical and numerical features were assembled into contigs, respectively. For the assembled *patient-specific* contigs, there were 20 of them with length ≥10,000 bp and all these contigs were matched to the *patient-specific* strain *B. thetaiotaomicron* VPI-5482 with 99.79–100% identity and 100% coverage. The coverage rate here means the proportion of contig sequence mapped to the strain. In contrast, these contigs cannot be matched to *B. thetaiotaomicron* 7330, and the maximum common sequences between contigs and *B. thetaiotaomicron* 7330 genome were no longer than 47 bp. For assembled *control-specific* contigs, there were 24 of them with length ≥5000 bp and all of them were mapped to the differentially abundant genome *B. caccae* with 100% identity and 100% coverage using BLAST (Altschul et al., 1997).

To evaluate the effect of *k*-mer length, we ran MetaGO on *10*-mer, *20*-mer, *30*-mer, *50*-mer, and *60*-mer, and the corresponding precision and recall are shown in **Table 2**. For the simulated dataset, When *k* = 10, no *group-specific* logical *k*-mers were identified. The recall rates for the identified numerical *k*-mers were only 25.34% for *B. caccae* ATCC 43185 and 22.45% for the common regions between *B. thetaiotaomicron* VPI-5482 and *B. thetaiotaomicron* 7330. When *k* ≥ 20, the effects of the *k*-mer length on the performance of our methods were small. The precision increased slightly with the *k*-mer length from 99.03 to 99.35% for logical *k*-mers and from 96.81 to 98.58% for numerical *k*-mers, consistent with the intuition that long *k*-mers can capture more specific information of each group. On the other hand, though almost all the recall rates were all above 90%, the recall first increased with *k*-mer length until *k* = 40 and then decreased, which might be caused by insufficient coverage for long *k*-mers.

**Table 2:** The precision and recall of MetaGO for the simulated dataset using different *k*-mer lengths.

*k*-mer length		10 (%)	20 (%)	30 (%)	40 (%)	50 (%)	60 (%)
Logicalized *k*-mers	Precision	–^∗^	99.03	99.05	99.11	99.45	99.35
	Recall	–^∗^	89.79	92.16	98.89	97.01	95.23
Numercial *k*-mers	Precision	99.63	96.81	96.07	97.72	98.22	98.58
	Averaged recall	23.89	95.70	97.93	98.00	96.82	94.76

*The “averaged recall” in numerical k-mers is the average of the recall of B. caccae ATCC 43185 genome and the recall of the common regions between strain B. thetaiotaomicron 7330 and B. thetaiotaomicron VPI-5482. ^∗^When k = 10, there is no logicalized k-mer identified, so it is marked with “–”.*

The experimental results demonstrate that the identification of *group-specific 40*-mers can not only capture genomes with different abundance but also identify *group-specific* markers under the strain-level resolution. Even though the two strains *B. thetaiotaomicron* VPI-5482 and *B. thetaiotaomicron* 7330 share 87% common sequences, our method still captured the *group-specific* sequences.

### The LC-Associated Metagenomic Dataset

MetaGO was applied to the large-scale metagenomic LC-associated dataset (Qin et al., 2014). With sufficient training samples and long read length, the *k*-mer length was set as *k* = 40. A total of ∼10^9^ non-zero *40*-mers were found in the feature matrix of training samples. After removing the highly sparse *40-*mer features, ∼10^6^ features were left.

#### Identify *Group-Specific* Features

Using ASS > 0.8 as the threshold, 37,302 logical features were identified as *group-specific 40-*mers. That is, any one of these *40*-mers could achieve ASS > 0.8 using its corresponding *single-logical-feature* predictor on training samples. We then used each of these 37,302 *single-logical-feature* predictors to predict LC in the validation and testing sets. As shown in the histogram of **Figure 3A**, ASS values of validation and testing were centered at 0.85 and 0.78, respectively. Among the 37,302 *single-logical-feature* predictors, 35,404 (95%) *group-specific 40-*mers achieved ASS ≥ 0.8 on the validation set, and 12,750 (36%) achieved ASS ≥ 0.8 on the testing set. Furthermore, 345 numerical features were identified as *group-specific 40*-mers with ASS ≥ 0.8, where 248 and 194, respectively, achieved ASS ≥ 0.8 on validation and testing sets using corresponding *single-numerical-feature* logistic regression predictors. All 37,302 logical and 345 numerical *40*-mers were *LC-specific* in that they were all present only in the fecal samples of LC patients, but not in the samples from healthy controls. The identified *group-specific 40*-mers for the LC dataset are available in **Supplementary File S2**.

We also implemented a controlled trial by shuffling the labels of the training samples randomly. Using the same pipeline and settings, only 247 *40*-mers achieved ASS ≥ 0.7, and the highest value was 0.73. This control trial indicates that most of the identified *group-specific 40*-mers for LC were more likely to be true rather than due to false positives.

#### Classification With the *Group-Specific* 40-mer(s)

We used classification performance to evaluate the discriminative capability of the identified *group-specific 40*-mers. First, we classified the healthy and LC groups with single features. The *single-logical-feature* predictor that obtained the highest ASS = 0.87 on the training set achieved ASS = 0.885 (sensitivity = 0.81 and specificity = 0.96) on the validation set and 0.87 (sensitivity = 0.84 and specificity = 0.90) on the independent testing set. Second, we built a classifier using a set of features. Using the top 10 *group-specific 40*-mers, a random forests classifier achieved AUCs of 0.963 on training, 0.969 on validation, and 0.942 on testing sets, respectively. The corresponding ROC curves are shown in **Figure 3B**. As shown in **Table 3**, Qin et al. (2014) obtained AUC = 0.918, 0.838, and 0.836 on training, validation, and testing sets with SVM using 15 marker genes as features. Pasolli et al. (2016) obtained AUC = 0.946 ± 0.036 with random forests using 542 species-abundance features and 0.963 ± 0.027 with SVM using 91,756 strain-specific markers features over 20 independent runs of 10-fold cross-validations, where cross-validations gave much more optimistic results, and many more features were adopted. The experiments show that *group-specific 40*-mers achieved better classification performance with fewer features.

**Table 3:** Comparison of the prediction performance of different methods based on the LC dataset.

Feature		*40*-mer	*40*-mer	Gene markers^††^	Species abundance^†^	Presence of strain- specific markers^†^
Experiment			Training (66P+56H) Validation (32P+27H) Testing (25P+31H)		20 runs of 10-fold cross-validation (114P+118H)	
Number of feature		**1**	**10**	15	542	120553
Classifier		**Single** **logical** **feature** **predictor**	**Random** **forests**	Support vector machine	Random forests	Support vector machine
AUC	Training validation testing	**ASS^∗^ = 0.87** **ASS = 0.885y** **ASS = 0.87**	**0.963** **0.969** **0.942**	0.918 0.838 0.836	0.946 ± 0.035	0.963 ± 0.027

*Using much fewer features, MetaGO achieved better results compared to other methods. The results of MetaGO were in bold. ^†^(Pasolli et al., 2016); ^††^(Qin et al., 2014); ^∗^average of sensitivity and specificity.*

#### *Group-Specific* Sequences

**FIGURE 4:**
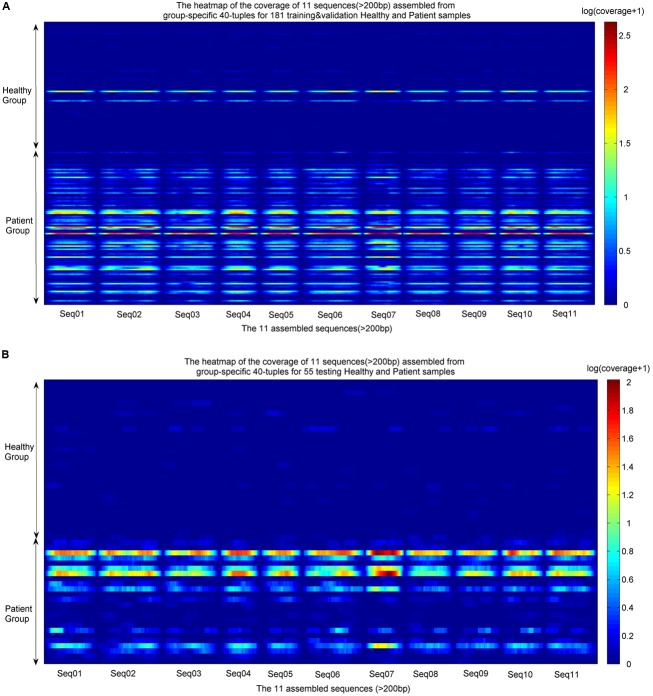
Heatmaps of coverage distribution over the 11 assembled sequences by the metagenomic reads from the training, validation, and testing samples. **(A)** Heatmap of the reads coverage of the 11 assembled sequences across the training and validation samples (83 healthy individuals and 98 LC patients). **(B)** Heatmap of the reads coverage of the 11 assembled sequences across the testing samples (30 healthy individuals and 25 LC patients). The coverage is the read-alignment depth in each nucleotide normalized by the number of million reads. To avoid the effect of large span, we use the logarithm of (coverage+1) as the numerical value of the heatmaps. The horizontal axis is composed of each nucleotide of the 11 sequences, and the vertical axis is composed of healthy individuals and patients. The upper part of each heatmap is the healthy group, and the lower part is the patient group.

The identified *group-specific 40*-mers were assembled into *group-specific* sequences using CAP3 (Huang and Madan, 1999), in which 11 assembled sequences were longer than 200 bp, with length from 210 to 350 bp (available in **Supplementary File S2**). They were aligned by the sequencing reads from the training and validation sets and the independent testing sets. The coverage distributions over the 11 sequences across all samples were represented as heatmaps in **Figure 4**. A noticeable difference appears between the two groups. In the group of healthy individuals, the reads of most samples cannot be aligned to the 11 sequences. In the patient group, the 11 sequences were aligned successively by the reads from most patients. The *de novo* and reference-free assembly produces longer *group-specific* sequences, which enables the discovery of biomarkers.

#### Taxonomic Information of the *Group-Specific* Markers

We aligned the 11 *LC-specific* sequences to genomes with “Nucleotide Blast” in NCBI, and all of the sequences were aligned to two strains of *V. parvula*, UTDB1-3, and DSM2008, with 100% query coverage and 97–100% identity. In a previous analysis based on the alignments from reads to reference genomes (Qin et al., 2014), *V. parvula* demonstrated a significant difference in abundance between the two groups of LC patients and healthy individuals.

**FIGURE 5:**
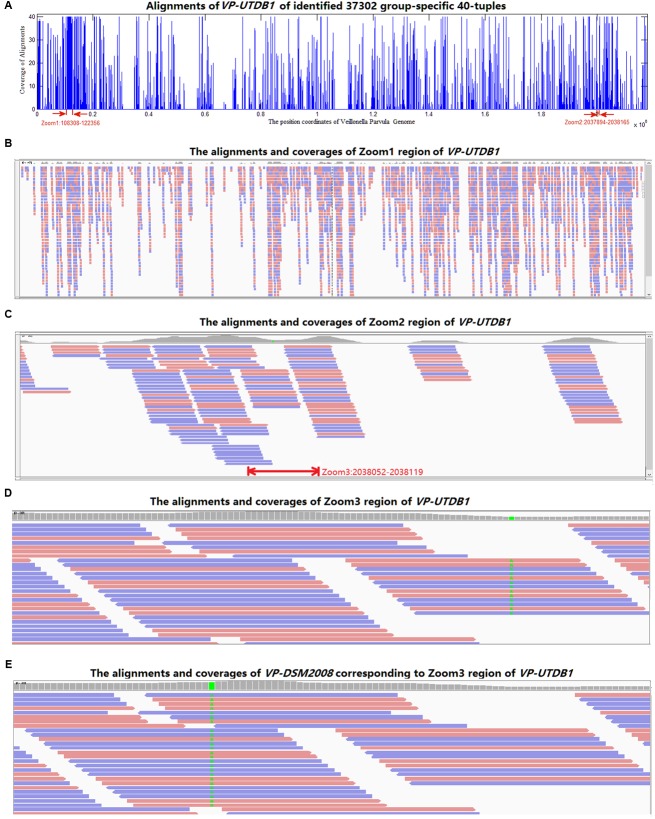
The alignments of the identified *group-specific 40*-mers to the genome sequence of *V. parvula* strain UTDB1-3. **(A)** The alignment distribution over the whole genome. **(B)** The alignments and coverages of region 108,308–122,356 (Zoom1). The red and blue bars denote the *40*-mers matched to reference genome sequence forward and backward, respectively. **(C)** The alignments and coverages of region 2,037,894–2,038,165 (Zoom2). **(D)** The alignments and coverages of region 2,038,053–2,038,119 (Zoom3) with consensus mismatches on 2,038,082. **(E)** The alignments and coverages of region 1,423,893–1,423,993 of *V. parvula* strain DSM2008. This region corresponds to the Zoom3 region of *V. parvula* strain UTDB1-3. Comparing the two regions in the two strains, the consensus mismatch (in green color in **D**) on UTDB1 is absent on DSM2008, but DSM2008 presents another consensus mismatch (in green color in **E**) on DSM2008: 1,423,924.

All 37,302 *group-specific* logical features and 345 *group-specific* numerical features were also blasted to reference genomes in NCBI, 31,067 of logical and 268 of numerical *40*-mers could be matched to *V. parvula* strain UTDB1-3, and 29,712 of logical and 262 of numerical *40*-mers could be matched to *V. parvula* strain DSM2008. Using *V. parvula* strain UTDB1-3 as an example, **Figure 5A** shows the coverage of the whole genome (2.17 Mbp) by the *LC-specific 40*-mers. The horizontal axis is the whole genome. The *40*-mers covered most parts of the genome. **Figures 5B–D** are the zoomed-in alignments and coverages of the genome: 108,308–122,356, 2,037,894–2,038,165, and 2,038,052–2,038,119, marked as “zoom1,” “zoom2,” and “zoom3”, respectively, in the figure. It is clear that many regions are highly and consecutively covered by *k*-mers. As shown in **Figure 5E**, region 1,423,893–1,423,993 of *V. parvula* strain DSM2008 corresponds to “Zoom3” region of *V. parvula* strain UTDB1-3. Comparing the regions in these two strains, the consensus mismatch against UTDB1 is absent on DSM2008, while DSM2008 presents another consensus mismatch against DSM2008: 1,423,924. The consistent mismatches against strains UTDB1 and DSM2008 in *V. parvula* indicate the possible existence of an unknown strain of *V. parvula*, which would exist in the gut of LC patients but be absent in the gut of healthy controls.

### The IBD-Associated and WT2D- Associated Metagenomic Datasets

The additional two disease-associated metagenomic datasets were analyzed with 20 independent runs of 10-fold cross-validation to evaluate the classification performance for easy comparison with previous studies. We emphasized that feature preprocessing and selection were done using only the training set, thereby avoiding biased and overly optimistic performance (Zhang et al., 2006; Pasolli et al., 2016).

#### The IBD-Associated Dataset

**FIGURE 6:**
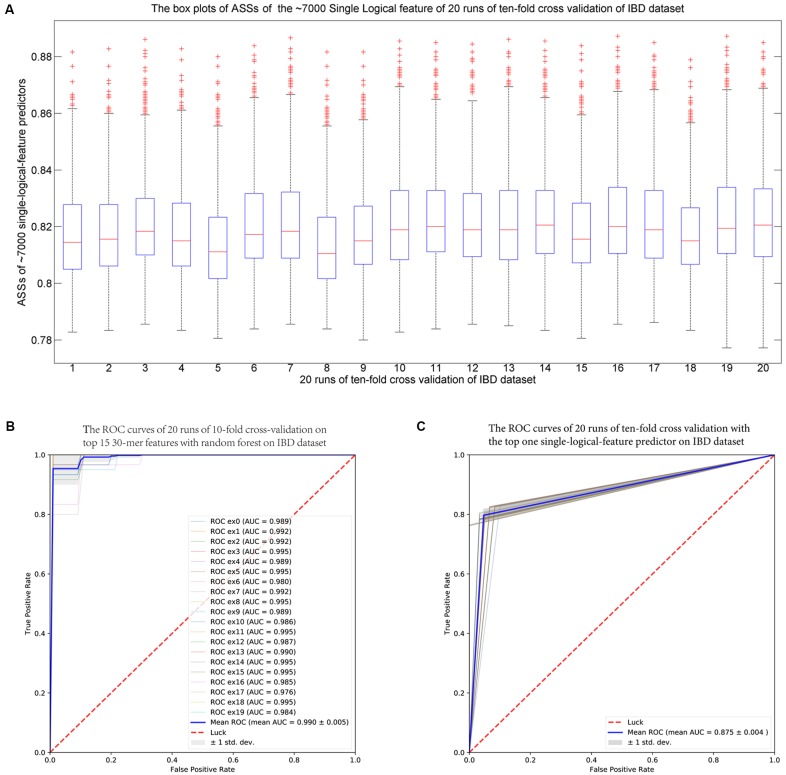
**(A)** The IBD-associated dataset: the boxplots of ASS by *single-logical-feature* predictors on each one of the identified ∼7000 *group-specific* features in the 20 independent runs of 10-fold cross-validation on the IBD dataset. Each boxplot is composed of ∼70,000 ASS values on each round of cross-validation. The ASS values are between 0.78 and 0.89 and centered on 0.81–0.82. The “+” symbol denotes outliers. **(B)** The ROC curves of the IBD-associated dataset: The top 15 ranked *30*-mers were combined to design the random forests classifier. The 20 ROC curves are from the 20 independent runs, and each one is the average over the 10-folds of cross-validation. The mean AUC is 0.990 ± 0.005 (95% confidence interval). **(C)** The ROC curves of the WT2D-associated dataset: The top 10 ranked *40*-mers were combined to design the random forests classifier. The 20 ROC curves are from the 20 independent runs, and each one is the average over the 10-folds of cross-validation. The mean AUC is 0.939 ± 0.011 (95% confidence interval).

For each fold test of 10-fold cross-validation, about 7000 *group-specific* logical features with ASS ≥ 0.8, but no *group-specific* numerical features, were identified. The numbers of *group-specific* features varied with different fold tests. Because of the relatively small sample size, *30*-mers were set as features. For each *group-specific 30*-mer, its *single-logical-feature* predictor yielded an ASS score on validation. For each round of cross-validation, ∼7000×10 (∼7000 *single-logical-feature* predictors and 10-folds) ASS values were obtained on validations. The boxplots in **Figure 6A** present the distribution of the ∼70,000 ASS values in 20 rounds of 10-fold cross-validation. The values are between 0.78 and 0.89, and they centered at 0.81–0.82, indicating that individual binary features can achieve ASS ≥ 0.78 solely on validation. The average ASS score is 0.875 ± 0.004 (95% confidence interval). The top 15 ranked features were combined to design a random forests classifier. **Figure 6B** presents the ROC curves of 20 independent runs, which were averaged over the 10-folds of cross-validation. The mean AUC of 20 runs is 0.990 ± 0.005 (95% confidence interval), which is much higher than the results reported in previous studies. As shown in **Table 4**, using the same dataset, Pasolli et al. (2016) designed two classifiers. The random forests classifier based on 443 species-abundance features achieved an averaged AUC = 0.893 ± 0.080 under the same experimental setting. The SVM classifier based on the presence of 91,756 strain-specific markers achieved AUC = 0.914 ± 0.084. Xing et al. (2017) obtained AUC = 0.967 with a logistic regression model with LASSO penalty in leave-one-out cross-validation (LOOCV), which used the relative abundances of bins as features. In another study, Cui and Zhang (2013) obtained accuracy = 88%, sensitivity = 92%, and specificity = 84% with 200 *7*-mer features at LOOCV on 25 healthy subjects and 25 patients, where the samples were the subset of our experiment and LOOCV was more relaxed than 10-fold cross-validation.

**Table 4:** Comparison of performance of different methods based on the IBD and WT2D datasets.

	IBD dataset						
Experiment			20 runs of 10-fold cross-validation (25P+97H)				Five runs of LOOCV (25P+25H)
**Feature**	***30*-mer**		***30*-mer**	Species abundance^†^	Presence of strain-specific markers^†^	Abundance in contig bin^††††^	*7*-mer^††^
**Number of feature**	**1**		**15**	443	91756	Not mentioned	200
**Classifier**	**Single** **logical** **feature** **predictor**		**Random** **forests**	Random forests	Support vector machine	Logistic regression + LASSO	Support vector machine
**AUC**	ASS* = 0.875 ± 0.004		**0.990 ± 0.005**	0.893 ± 0.080	0.914 ± 0.084	0.967	Accuracy = 0.88

	**WT2D dataset**						
**Experiment**			**20 runs of 10-fold cross-validation (52P +43H)**			**Training (20H+20P)** **Testing (32P+13H)**	

**Feature**	***40*-mer**	***40*-mer**	Species abundance^†^	Presence of strain-specific markers^†^	Gene markers^†††^	Abundance of bins with MetaGen	***40-mer***
**Number of feature**	**1**	**10**	381	83456	50	3	**3**
**Classifier**	**Single** **logical** **feature** **predictor**	**Random** **forests**	Random forests	Support vector machine	Support vector machine	Random forests	**Random** **forests**
**AUC**	**ASS = 0.76 ± 0.003**	**0.939 ± 0.011**	0.772 ± 0.116	0.785 ± 0.104	0.83	0.961 (training) 0.685 (testing)	**0.979 (training)** **0.782 (testing)**

*Using much fewer features, MetaGO achieved better results compared to other methods. The results of MetaGO were in bold. There were two experimental setting for IBD dataset, the “Five runs of LOOCV” are the subset of our experiment and LOOCV was more relaxed than 10-fold cross-validation. For the WT2D dataset, *40*-mers were tested under two experimental setting for comparing with other methods. ^†^(Pasolli et al., 2016); ^††^(Cui and Zhang, 2013); ^†††^(Qin et al., 2014); ^††††^(Xing et al., 2017); ^∗^average of sensitivity and specificity.*

#### The WT2D-Associated Dataset

For each fold test of 10-fold cross-validation, ∼700 *40-*mers with ASS ≥ 0.75 were identified, and the best ASS score was 0.78. The classifier designed with random forests using 10 top *group-specific 40*-mer features obtained an average AUC = 0.939 ± 0.011 on the 20 independent runs of 10-fold cross-validation, as shown in **Figure 6C**. In previous studies under the same experimental setting, the average AUCs were 0.834 using 50 metagenomic clusters as features (Karlsson et al., 2013) and 0.785 ± 0.104 using the presence of 83,456 strain-specific markers as features (Pasolli et al., 2016). For further comparison, we implemented metagenome-wide *de novo* assembly with MegaHIT (Li et al., 2015) and then binned the contigs with MetaGen (Xing et al., 2017). The relative abundances of bins were used as features to separate the patient and control groups. The total of 96 samples were too large for read assembly, which required >256 GB memory for 80 samples, and the alignments of reads to the contigs were time-consuming. Therefore, 20 patients and 20 healthy individuals were randomly selected as the training set. The remaining 56 samples were used for independent testing. The relative abundances of bins generated by MetaGen were used as features and the random forests classifier was designed on the training set. The definition of relative abundance in MetaGen includes the parameters that should be determined for each species (they assumed each bin is each species) and each sample through the algorithm of MetaGen. When the classifier was tested on the independent set, these parameters for independent samples are also required to be determined. Personal communications with MetaGen’s developers, we revised the code of MetaGen and calculated the feature values of the relative abundances of selected bins for each testing sample. With random forests, MetaGen achieved AUC = 0.685 using 3 features of bins and AUC = 0.735 using 15 features of bins on testing data. With the same training samples, our pipeline obtained AUC = 0.782 with 3 features of *k*-mers and AUC = 0.794 using 15 features of *k*-mers with random forests on testing data. Although both methods are reference free, the *group-specific k*-mers show greater discriminative power than the contig bins for predicting the disease status. Besides, the *de novo* assembly and contig binning are time-consuming. For example, it took 120 h to finish the running from read assembly to contig binning on this training set.

From the experiments, IBD is more predictable than T2D. The experiments on the two disease-associated datasets demonstrate that *group-specific k*-mers achieved much better classification performance with fewer features than previous studies that used the features of short *k*-mer frequencies, species abundance, and strain marker presence. The experiments confirm the effectiveness of long *k*-mer features and the strategy of identifying *group-specific* features.

### Running the Computational Pipeline on *Apache Spark*

For the LC dataset, it took 65 h to identify the *group-specific 40*-mers from 56 healthy and 66 LC training samples (252 GB fasta.gz files), including the calculation of *40*-mer frequency vector, the integration of feature matrix, and the identification of the *group-specific 40*-mers. The peak storage space is about 1.5 TB. The above result was run on a local mode of a server with 128 G-memory and Intel(R) Xeon(R) CPU E5-2620 v4 with 8 CPU cores at 2.10 GHz.

## Discussion

Different diseases have different levels of association-complexity with human microbiome. If one disease is significantly associated with a specific microbial strain/species/gene, then the disease is highly predictable using a *single-feature* predictor. That is, the disease can be diagnosed with a single microbial biomarker. However, many human diseases are complex in the sense that multiple *group-specific* markers are required to characterize the relevance of disease and microbiome. For these diseases, we have shown that combining several *group-specific* features can improve prediction accuracy.

In MetaGO, features were selected based on three preset thresholds, including ASS of *single-logical-feature* predictor (𝜃_1_), *p*-value of Wilcoxon rank-sum test for numerical features (𝜃_2_), and *single-numerical* logistic-regression predictor (𝜃_3_). For the IBD-associated and LC-associated datasets, we set 𝜃_1_ = 0.8, 𝜃_2_ = 0.01, and 𝜃_3_ = 0.8, respectively. However, for diseases having more complex associations with microbiome, such as T2D (Pasolli et al., 2016), 𝜃_1_ was relaxed to 0.75, 𝜃_2_ = 0.05 and 𝜃_3_ = 0.75. Therefore, the three thresholds were, in effect, set according to the expected discriminant power of features and the complexity of association between disease and microbiome.

MetaGO was designed and implemented for two-group case and control datasets. For some studies, there may exist multiple subgroups for the disease, or a pre-disease group. An example of subgroups for disease is the AR-type (marked akinesia and rigidity) and T-type (predominant resting tremor) in Parkinson’s disease (Paulus and Jellinger, 1991). Two examples of pre-disease state are impaired glucose tolerance state between T2D and normal glucose tolerance (Karlsson et al., 2013) and colorectal adenoma state between carcinoma and healthy state (Feng et al., 2015). For the multiple-groups scenario, the way to use MetaGO depends on the research purpose. If the purpose is to identify some microbial organisms that are associated with all sub-groups of the disease, we can combine all individuals belonging to any disease groups and treat them as one disease group. MetaGO can be used to the disease and control groups to identify the common microbial organisms associated with all groups of diseases. On the other hand, if the purpose is to identify certain microbial organisms that are specific to a particular group, we can combine all other individuals into one group and then use MetaGO to identify group-specific-associated microbial organisms. Extending MetaGO for a joint analysis of *group-specific* organisms in all the control and different disease groups is a topic of further study.

## Conclusion

In this study, we developed a computational framework, MetaGO, that is free from reference sequences, metagenome-wide *de novo* assembly, and sequence alignment, to identify *group-specific* sequences between two groups of microbial communities using long *k*-mer features. The *k*-mer length was set between 30 and 40 based on the tradeoff among sensitivity, specificity, and computational cost. The identified *group-specific k*-mers present improved discriminant power for diagnosing diseases using human gut metagenomics data compared with previous studies.

To overcome the computational challenge of long *k*-mer features, an open-source, parallel-computing pipeline was developed on *Apache Spark* to save computational resources and reduce running time. In this study, we applied MetaGO to analyze metagenomic disease-associated datasets. It should be noted that the pipeline is also suitable for identifying *group-specific k*-mers for all types of high-throughput sequencing data where samples are collected from different groups, such as disease-associated human genome sequencing data or other phenotype-associated metagenomic datasets from different environments.

Our experiments validated improvements made by the identified *group-specific k*-mer features compared to previous studies using other types of features. The *group-specific* sequences offer deep and detailed insights required to understand the differences between groups because the method essentially identifies a sequence that is present, or rich, in one group, but absent, or scarce, in another group, the fundamental working principle of *group-specific* sequences. We found that biological explorations based on *group-specific* sequences are consistent with those from previous biological experiments, but additionally offered the potential for new discoveries. Therefore, using long *k*-mer sequence signatures is an effective way to discover biological features, paving the way for a new paradigm of biomarker discovery in the context of host phenotypes. MetaGO enables the detection of *group-specific* features and development of prediction models using a single feature, or a combination of a few features, which helps to reduce the complexity of the model, while increasing the potential feasibility of follow-up discovery of discriminative microbial biomarker(s) for the easy diagnosis of human diseases.

## Availability of Supporting Data and Source Codes

Source codes and testing data are available at https://github. com/VVsmileyx/MetaGO. The metagenomic sequencing datasets of IBD, LC, and T2D of European women were from the European Bioinformatics Institute’s European Nucleotide Archive under accession numbers (EMBL: ERP000108, ERP005860, and ERP002469).

## Author Contributions

YW, FS, and TC planned the project. YW and ZY designed the model and experiments. LF performed the experiments. YW, JR, and FS analyzed the data. LF contributed materials/analysis tools. YW, JR, ZY, and FS wrote the main manuscript. All authors read and approved the final manuscript.

## Conflict of Interest Statement

The authors declare that the research was conducted in the absence of any commercial or financial relationships that could be construed as a potential conflict of interest.

## Supplementary Material

FILE S1 Detailed descriptions of method and results.

FILE S2 *LC-specific 40*-mers and sequences.
