# Supplementary material for: Identifying Group-Specific Sequences for Microbial Communities Using Long k-mer Sequence Signatures
Source: Front Microbiol. 2018 May 3;9:872. doi: 10.3389/fmicb.2018.00872 (PMC5943621; doi:10.3389/fmicb.2018.00872)
Supplement: FILE S1 — Detailed descriptions of method and results. [file Presentation_1.pdf]

Supplementary 1: Some detail descriptions of methods

1. An example of a numerical feature, a logical feature, a single-logical-feature predictor, a single-numerical logistic regression predictor and a group-specific feature.

For *40*-mer "AAACCGCACACCATCCATTGGGTGTCTGGCAATCAAGTCA", the **numerical feature** on 20 healthy individuals and 20 patients is:

$$F_{\mathrm{AAACCGCACACCATCCATTGGGTGTCTGGCAATCAAGTCA}}(\mathrm{H1} \cdots \mathrm{H20};\ \mathrm{P1} \cdots \mathrm{P20})$$

$$= (0.1\ 0.1\ 0.2\ 0.2\ 0.3\ 0.1\ 0.1\ 0.3\ 0.1\ 0.1\ 0.2\ 0\ 0.04\ 0.05\ 0.04\ 0\ 0.1\ 0.1\ 0\ 0.1;\ 0\ 0\ 0\ 0\ 0\ 0\ 0\ 0\ 0.3\ 0\ 0\ 0\ 0\ 0\ 0.2\ 0\ 0\ 0\ 0\ 0).$$

The corresponding **logical feature** is:

$$F^{l}_{\mathrm{AAACCGCACACCATCCATTGGGTGTCTGGCAATCAAGTCA}}(\mathrm{H1} \cdots \mathrm{H20};\ \mathrm{P1} \cdots \mathrm{P20})$$

$$= (1\ 1\ 1\ 1\ 1\ 1\ 1\ 1\ 1\ 1\ 1\ 0\ 1\ 1\ 1\ 0\ 1\ 1\ 0\ 1;\ 0\ 0\ 0\ 0\ 0\ 0\ 0\ 0\ 1\ 0\ 0\ 0\ 0\ 0\ 1\ 0\ 0\ 0\ 0\ 0)$$

For the logical feature, the sensitivity and specificity of the ***single-logical-feature*** **predictor** on the training sample $\mathrm{H1} \cdots \mathrm{H20};\ \mathrm{P1} \cdots \mathrm{P20}$ were 0.90 and, 0.85 respectively. For the numerical feature, its ***single-numerical-feature*** **logistic-regression predictor** is $\mathrm{P}(y = 1) = \frac{1}{1+e^{14.6x-0.84}}$ and the corresponding sensitivity and specificity for training set are 0.90 and 0.70, respectively.

If using $\frac{(sn+sp)}{2} \geq 0.8$ as threshold, 40-mer AAACCGCACACCATCCATTGGGTGTCTGGCAATCAAGTCA is **a *group-specific* feature**.

2. The software implementation on Apache Spark

Apache Spark is a fast big data analytics engine on MapReduce framework and Hadoop Distributed File System (HDFS). In parallel programming, *Spark* supplies Resilient Distributed Datasets (RDDs) and parallel operations on datasets. RDD enables programmers perform in-memory computations on large clusters. The map and reduce RDD frame works is shown in Figure S1.

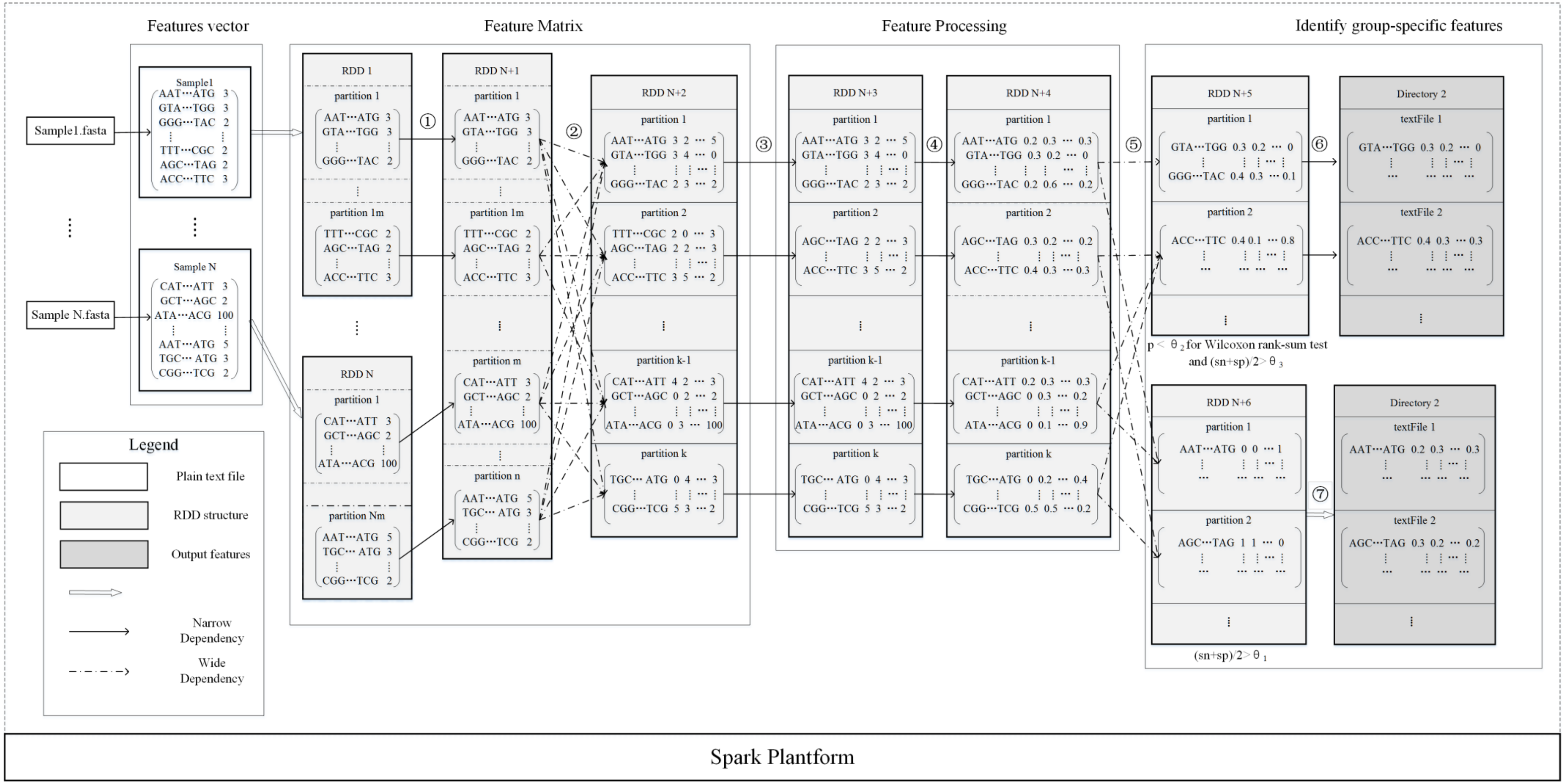

Figure S1 The RDD framework of the computation pipeline.

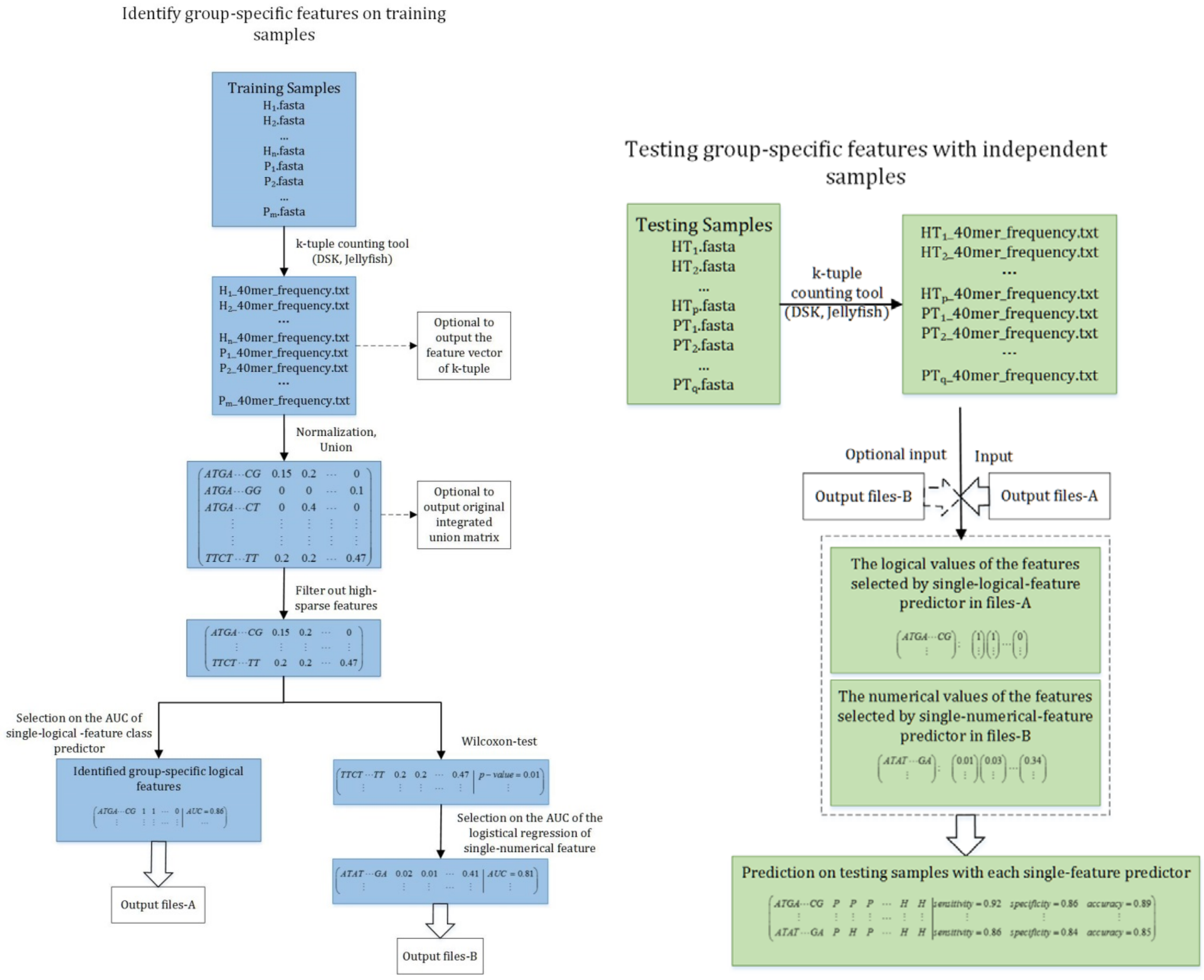

Figure S2 The implementation flows of identifying *group-specific* features and testing of the prediction performance of the group-specific features.
